# Borrelial lymphocytoma mimicking primary cutaneous follicular B-cell lymphoma in 2 adult patients – Diagnostic and therapeutic approach

**DOI:** 10.1016/j.jdcr.2025.02.015

**Published:** 2025-03-07

**Authors:** Anna Barbara Henricus Hompesch, Julia Maria Käthe Clabbers, Betül Cosgun, Elizabeth Morreel, Myrurgia Amira Abdul Hamid, Antoni Henryk Gostyński

**Affiliations:** a Department of Dermatology, Maastricht University Medical Centre+, Maastricht, the Netherlands; b Department of Medical Microbiology, Maastricht University Medical Centre+, Maastricht, the Netherlands; c Department of Pathology, Maastricht University Medical Centre+, Maastricht, the Netherlands

**Keywords:** Borrelia burgdorferi, borrelial lymphocytoma, cutaneous lymphoid hyperplasia, Lyme disease, primary cutaneous follicular B-cell lymphoma, pseudolymphoma

## Introduction

Borrelial lymphocytoma (BL) is a rare cutaneous manifestation of Lyme borreliosis and belongs to the group of cutaneous pseudolymphomas.^1^^,^^2^ It has many synonyms, such as Borrelia-associated nodular B-cell pseudolymphoma, -lymphocytoma cutis, and -cutaneous lymphoid hyperplasia. The distinction between primary cutaneous lymphomas and cutaneous pseudolymphomas can be very difficult, particularly in BL, but is of high importance due to differences in treatment and prognosis.^1^^,^^2^ In this case report, we will discuss 2 adult patients with BL of the areola, that were first suspected of primary cutaneous follicular B-cell lymphoma (PCFCL). Both patients responded very well to doxycycline 100 mg twice a day for 10 days.

## Case 1

**Fig 1:**
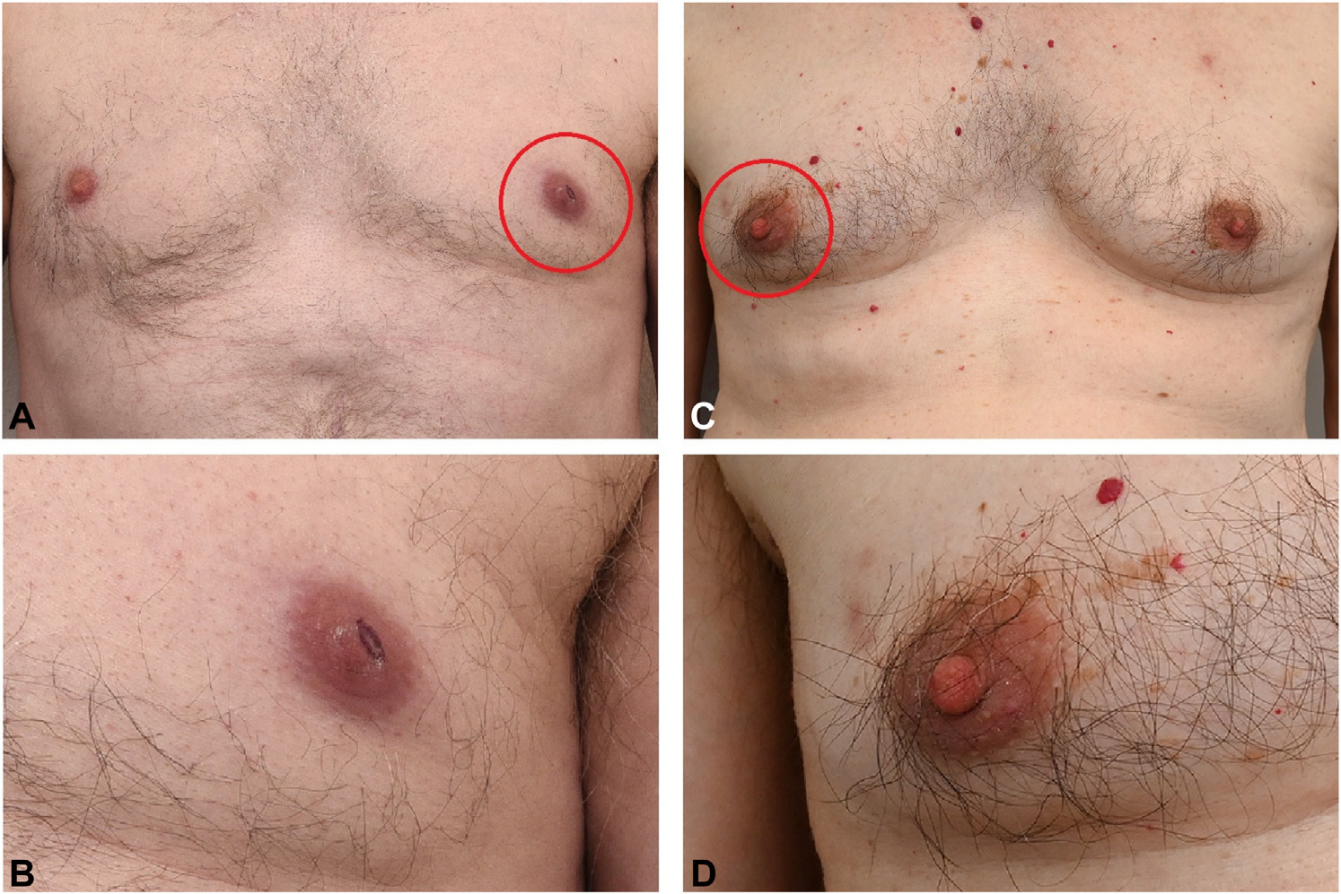
Borrelial lymphocytoma (BL) can be recognized as a bluish-red plaque with a diameter of 1 to 5 centimetres. In patient 1 (**A** and **B**), BL is located on the left areola, in patient 2 (**C** and **D**), BL is located on the right side. The affected areola is noticeably enlarged in both patients.

A 66-year-old male patient, working in the agricultural sector, presented with a painless infiltrated red plaque on the left nipple for 1 month (Fig 1). He had no B-symptoms. No tick bite could be recalled. Ultrasonography and mammography of the left breast detected no masses. Histopathologic findings were highly suggestive for PCFCL (Table I). Clinically, BL was added to the differential diagnosis, as the nipple is a common area for this disease. A computed tomography scan for staging of the head, neck, chest, and abdomen showed generalized lymphadenopathy without extracutaneous manifestations. Borrelia serology was positive with immunoglobulin M (IgM) and immunoglobulin G (IgG) immunoblot confirmation. *Borrelia burgdorferi* polymerase chain reaction (PCR) conducted on skin biopsy was negative. The patient was treated with doxycycline 100 mg twice a day during 10 days and complete resolution was achieved. No relapse was observed after a follow-up period of 12 months. We instructed the patient to come back in case of recurrent symptoms, he did not consult our department the past 5 years.

**Table I:** Overview of histological characteristics of our 2 cases compared to primary cutaneous follicular B-cell lymphoma (PCFCL), primary cutaneous marginal zone lymphoma (PCMZL), and borrelial lymphocytoma (BL)^2^, ^3^, ^4^

PCFCL	BL	PCMZL	Patient 1	Patient 2
Histology				
Follicular, diffuse or mixed pattern. Centrocytes and centroblasts	Follicular pattern with tingible body macrophages, Polymorphous; Lymphocytes, small-medium sized, plasmacells, histiocytes, eosinophils	Diffuse pattern. Polymorphous; Lymphocytes, small-medium sized (marginal zone-like), lymphoplasmacytic- and plasmacells	Follicular and diffuse pattern. Predominantly B-cells and several plasma cells	Follicular pattern, a dense infiltrate with atypical small and large B-cell Lymphocytes, plasmacells
Immunohistochemistry				
Bcl-6+	Bcl-6+	Bcl-2+	Bcl-6+	Bcl-6+
Bcl-2-	Bcl-2-	Bcl-6-	Bcl-2-	Bcl-2-
Irregular and not restricted to the germinal centers		Light chain restriction in lymphoplasmacytic/plasmacells may be present	No light chain restriction in plasmacells	No light chain restriction in lymphoplasmacytic/plasmacells
Molecular diagnostics				
Clonally rearranged Ig heavy and light chain genes	Polyclonal or clonally rearranged Ig heavy and light chain genes	Clonally rearranged Ig heavy and light chain genes or polyclonal (∗)	Clonally rearranged in a polyclonal background	Predominantly polyclonal with a weak clonal B-cell population

*BL*, Borrelial lymphocytoma; *PCFCL*, primary cutaneous follicular B-cell lymphoma; *PCMZL*, primary cutaneous marginal zone lymphoma.

∗ Clonal rearrangement can be missed due to low sensitivity in detecting neoplastic cells in an extensive reactive infiltrate.

## Case 2

A 75-year-old male patient presented with a painful infiltrated red plaque on the right areola and induration of the surrounding breast tissue swelling for 3 weeks (Fig 1). B-symptoms were absent. No reported history of tick bite. Ultrasonography and mammography showed subtle thickening of the areolar region. No mass was detected. The histopathology was suggestive of a PCFCL (Table I). *B. burgdorferi* PCR conducted on isolated DNA of a histological section was positive. Borrelia serology showed a positive IgG and IgM immunoblot. Therefore, the diagnosis of BL was more likely. The patient was treated with doxycycline 100 mg twice a day for 10 days and the lesion resolved completely. No relapse was observed after 2 months follow-up. We instructed the patient to come back in case of recurrent symptoms, now 4 years ago, and received no indication of recurrence until today.

## Discussion

Lyme disease is the most frequent tick-born disease in North-America and Europe.^5^^,^^6^ BL is defined as a cutaneous lymphoid hyperplasia (pseudolymphoma)^4^ with reaction to *Borrelia burgdorferi sensu lato*, primarily to the subspecies *Borrelia afzelii*, transmitted by Ixodes ricinus ticks.^6^^,^^7^ *Borrelia afzelii* is endemic in Europe.^7^ 0.3% to 3% of all clinically apparent Borrelia sp. infections in Europe manifest as a BL, most of them in children.^2^ On the contrary, in the United States, *B. burgdorferi sensu stricto* is the most common causative agent of Lyme borreliosis, transmitted by Ixodes scapularis and – pacificus ticks, which is less frequently associated with BL.^2^^,^^6^^,^^7^ Consequently, BL is only rarely reported in the United States. BL appears between 2 days and 6 months after an infected tick bite.^1^^,^^2^^,^^6^^,^^8^ BL usually manifests as a bluish-red plaque with a diameter of 1 to 5 centimeters and can be accompanied by local discomfort. In adults, it is mostly located on the areola (73,6%) and the earlobe (18,8%) and in few cases the scrotum, the anterior axillary fold, nose, arm or shoulder are affected (7.6%).^3^^,^^5^^,^^9^ In children it presents predominantly on the earlobe (84% to 88%).^8^ BL can be preceded by or occur in concordance with erythema migrans (EM) (25% to 72.2%),^2^^,^^9^ but is also seen after a first presentation of EM.^5^^,^^9^ Extracutaneous symptoms such as elevated temperature and regional lymphadenopathy in concordance with BL are rare (0.49% and 0.98%, respectively).^2^^,^^8^^,^^9^

The biopsies of our patients were highly suggestive for PCFCL (Table I). However, PCFCL mostly occurs as multiple (confluent) nodules in an area of the trunk or the head.^10^ BL belongs to the group of cutaneous lymphoid hyperplasia (pseudolymphomas) which exhibit reactive changes in histology resembling a lymphoma. In our 2 cases, the clinical presentation corresponded with the diagnosis of BL, and Borrelia serology was positive. Good treatment response on doxycycline in both patients confirmed BL diagnosis and highlights the importance of clinico-pathologic correlation in diagnosis of (pseudo) lymphomas. In Table I, typical histologic findings of PCFCL, primary cutaneous marginal zone lymphoma, and our 2 cases are compared with BL.

Positive IgM and/or IgG for *Borrelia burgdorferi* can be found in 70% to 95% of the cases at presentation. However, negative testing does not exclude BL.^2^ Sensitivity of IgM and/or IgG serologic testing is 50% in case of EM.^2^ In both our patients, IgM and IgG were positive and confirmed by immunoblot, suggesting a recent infection with *Borrelia burgdorferi s.l.* Sensitivity of borrelial identification through PCR can vary greatly between methods and/or different types of samples, with a reported sensitivity of 25% to 90% and a specificity of 100% on skin biopsies of EM.^2^ In one of our cases, *Borrelia burgdorferi* PCR was positive, which attributed to the diagnosis. Various antibiotic agents and treatment durations have been recommended in the literature (Table II). We chose treatment with doxycycline 100 mg twice daily for 10 days, following the Dutch guideline, despite other recommendations in the literature of a treatment duration of 14-21 days. The good clinical response in both patients supports a shorter treatment duration of 10 days as advised in the Dutch guideline.

**Table II:** Therapeutic advice of several national guidelines for borrelial lymphocytoma^2^^,^^3^^,^^7^

Country	Guideline	1st choice therapy	2nd choice therapy	3rd choice therapy	4th choice therapy
The Netherlands, 2013^2^	Centraal Begeleidings-Orgaan (CBO) Guideline	Doxycycline 100 mg twice daily, 10 d	Amoxicillin 500 mg 3 times daily, 14 d	Azithromycin 500 mg once daily, 5 d	
Germany, 2017^7^	Guideline of the German Dermatology Society (DDG)	Doxycycline 100 mg twice daily or 200 mg once daily, 14-21 d	Amoxicillin 500-1000 mg 3 times daily, 14-21 d	Cefuroxime axetil 500 mg twice daily, 14-21 d	Azithromycin 250 mg twice daily, 5-10 d
United States of America, 2020^3^	Guideline of Infectious Disease Society of America (IDSA)	Doxycycline 100 mg twice daily, 14 d	Amoxicillin 500 mg 3 times daily, 14 d	Cefuroxime axetil 500 mg twice daily, 14 d	Azithromycin 500 mg once daily, 5-10 d

In conclusion, the discussed cases show possible difficulties in diagnosis of BL, as histopathologically it may mimic other lymphomas, the most common primary cutaneous marginal zone lymphoma, but also PCFCL. Therefore, the correlation of clinical presentation, with histopathological and serologic findings is crucial in the diagnosis of pseudolymphoma.^2^ PCR analysis of lesional skin on borrelia is less sensitive, but can be supportive in diagnosis. Doxycycline, amoxicillin, azithromycin, and cefuroxime seem to be superior therapy methods in BL. The precise duration of the antibiotic regimen is a remaining topic of discussion in literature. Our cases support the Dutch Centraal Begeleidings-Orgaan guideline advice of doxycycline 100 mg twice daily for 10 days.

## Conflicts of interest

None disclosed.
